# A Preliminary Study of Modulen IBD Liquid Diet in Hospitalized Dogs with Protein-Losing Enteropathy

**DOI:** 10.3390/ani12121594

**Published:** 2022-06-20

**Authors:** Aarti Kathrani, Gina Parkes

**Affiliations:** Department of Clinical Science and Services, Royal Veterinary College, Hertfordshire AL9 7TA, UK; gparkes@rvc.ac.uk

**Keywords:** nutrition, canine, gastrointestinal, diarrhea

## Abstract

**Simple Summary:**

Modulen IBD is an oral liquid food that results in remission rates similar to immunosuppressive drugs in children with inflammatory bowel disease. This diet has not been previously investigated in dogs. We aimed to describe the use of Modulen IBD in hospitalized dogs with inflammatory protein-losing enteropathy (PLE) when combined with whey powder and a multivitamin/mineral blend to ensure this was complete and balanced for dogs. Five dogs hospitalized for PLE that had an esophagostomy feeding tube placed were eligible and prospectively enrolled. All dogs received Modulen IBD without concurrent immunosuppressive drugs and tolerated tube feedings. All dogs had resolution of anorexia, three had stable or improved serum albumin concentrations, four had improved or normalized serum globulin concentrations, and four dogs had improved or normalized serum cholesterol concentrations 2–3 days after initiating the diet. In conclusion, the Modulen IBD liquid diet was well-tolerated in-hospital and resolved anorexia in all dogs and helped to improve selected biochemical parameters in some dogs. Further studies are needed to assess the long-term effects of feeding this diet to dogs with inflammatory PLE.

**Abstract:**

Modulen IBD is an enteral liquid diet that can induce remission rates similar to glucocorticoids in children with inflammatory bowel disease. The Modulen IBD liquid diet has not been previously investigated in dogs. Our study aimed to describe the use of the Modulen IBD liquid diet in hospitalized dogs with inflammatory protein-losing enteropathy (PLE), including its tolerance and effects on appetite and gastrointestinal signs, and laboratory parameters during hospitalization. Of the 14 dogs hospitalized for PLE that had an esophagostomy feeding tube placed at the time of endoscopy, 5 were eligible and prospectively enrolled. The Modulen IBD liquid diet was supplemented with whey powder isolate and a multivitamin/mineral blend to ensure the diet was complete and balanced for canine adult maintenance and had a macronutrient profile desirable for PLE. All five dogs tolerated tube feedings with the Modulen IBD liquid diet, allowing an increase of 75 to 100% of the resting energy requirement (RER) by day 3 to 4. The diet was administered without glucocorticoid in all five dogs. All five of these dogs had a resolution of anorexia allowing the voluntary intake of a commercial hydrolyzed protein diet prior to the use of glucocorticoids. Of these five dogs, three (60%) had stable or improved serum albumin concentrations (median % increase: 10.3, range: 0–31.1), four (80%) had improved or normalized serum globulin concentrations (median % increase: 12.9, range: 5.1–66.2) and four (80%) had improved or normalized serum cholesterol concentrations (median % increase: 31.5, range: 4.8–63) 2–3 days after initiating the diet. However, there were no significant differences in these selected biochemical parameters pre- and post-feeding with the diet (*p* > 0.080). In conclusion, the Modulen IBD liquid diet, fed via an esophagostomy feeding tube was well-tolerated in-hospital and resolved anorexia in all dogs and helped to improve selected biochemical parameters in some dogs. Further studies are needed to assess the long-term effects of feeding this diet on the rate of serum albumin increase and remission in dogs with inflammatory PLE.

## 1. Introduction

Approximately 50% of dogs with protein-losing enteropathy (PLE) due to chronic enteropathy (CE) or lymphangiectasia have a poor prognosis due to failure to respond to standard treatments, including immunosuppressive therapy [1,2]. It is therefore critical to develop more efficacious treatments for the management of dogs with inflammatory PLE. Exclusive enteral nutrition alone has been shown to induce remission in 80% of pediatric patients with Crohn’s disease (CD) [3], a form of human inflammatory bowel disease, and similar remission rates have been seen in adults with newly diagnosed CD [4]. Indeed, several studies and a meta-analysis comparing exclusive enteral nutrition to corticosteroids in pediatric patients with active CD found no significant difference in remission rates between the two forms of therapy [5,6,7]. Although the mechanism of action of exclusive enteral nutrition is unknown, it has been shown to improve mucosal healing (74% versus 33% with corticosteroids [8]), decrease inflammatory cytokines [9], alter the gut microbiota [10], improve nutritional status, and increase serum albumin concentrations compared to corticosteroids [11,12]. To our knowledge, exclusive enteral nutrition with a liquid diet has never been investigated in canine gastrointestinal diseases. As these diets result in greater intestinal mucosal healing and serum albumin concentrations compared to treatment with corticosteroids, studies assessing their effects in dogs with inflammatory PLE are warranted to determine if they can help to improve the response to treatment and prognosis of these dogs.

The Modulen IBD liquid diet is a powdered formulation that is used for the dietary management of active CD, as a sole source of nutrition or in the remission phase of CD, as a nutritional support. The diet can be administered as an oral sip feed or via an enteral feeding tube. The diet is 100% casein-based, using whole protein, and is supplemented with medium-chain triglycerides and carbohydrates in the form of glucose syrup, resulting in low osmolality for good tolerance. The diet contains transforming growth factor beta, a naturally occurring anti-inflammatory factor. The Modulen IBD liquid diet has been shown in multiple studies to decrease intestinal inflammation and promote mucosal healing in humans with CD [13,14,15,16]. To our knowledge, this diet has never been investigated in dogs. 

Our study aimed to describe the use of the Modulen IBD liquid diet fed exclusively via an esophagostomy tube in hospitalized dogs with inflammatory PLE. We aimed to determine the tolerance to this diet, its effects on appetite and gastrointestinal signs, and selected laboratory parameters during hospitalization in these dogs. Determining the short-term effect of this diet in dogs with inflammatory PLE will help to provide data to justify the potential cost and complexity of longer-term studies utilizing this diet. We hypothesized that exclusive enteral nutrition with the Modulen IBD liquid diet in hospitalized dogs with inflammatory PLE would be well-tolerated and result in improvement in appetite, gastrointestinal signs, and select biochemical parameters, including serum albumin, globulin, and cholesterol concentrations, during the hospitalization period. 

## 2. Materials and Methods

### 2.1. Eligibility Criteria

This study was performed at the Queen Mother Hospital for Animals, Royal Veterinary College, U.K from January 2020 to October 2020, inclusive. Dogs presenting to the Internal Medicine Service with gastrointestinal signs that had biochemical results consistent with PLE (panhypoproteinemia with low or low-normal serum cholesterol concentration) were eligible for inclusion, provided they had thorough and appropriate investigations to rule out all other known causes, such as hypoadrenocorticism and gastrointestinal parasites. Gastrointestinal histopathologic diagnosis and clinical indication for the placement of an esophagostomy feeding tube (the presence of anorexia for at least 3 to 5 days) was also required for inclusion in the study. Exclusion criteria included the presence of concurrent disease that may require additional dietary strategies, such as pancreatitis and hepatic or renal disease, if the dog was growing, pregnant or lactating, and gastrointestinal histopathology that subsequently showed neoplasia or changes consistent with infection rather than chronic inflammation. 

The dogs were enrolled at the time of gastrointestinal endoscopy and placement of an esophagostomy feeding tube. The dogs were treated with the Modulen IBD liquid diet without the use of concurrent glucocorticoids. All dogs were started on the treatment the same day after the collection of gastrointestinal biopsy specimens via endoscopy, and treatment was continued until the histopathology results were returned 3 to 5 days later. 

### 2.2. Formulation of the Diet

The diet was specifically formulated for each dog by a board-certified veterinary nutritionist (A.K.), taking into consideration the dog’s body weight at admission, biochemical results including electrolytes and cholesterol, and diet history including fat tolerance, using a computer software program (www.BalanceIT.com, accessed dates: January 2020 to October 2020). The diet was formulated to ensure it was complete and balanced and that all essential nutrients met the minimum requirements and/or recommended allowances per the National Research Council Nutrient Requirements 2006 for canine adult maintenance on an energy basis. 

As the Modulen IBD liquid diet is formulated for humans and not for dogs, two additional ingredients were required to be added to this diet to make it suitable for canine feeding. Firstly, whey powder was required to ensure that the diet met the protein requirements for canine adult maintenance and that the fat content did not exceed 35% on a metabolizable energy basis, and secondly, a multivitamin and mineral blend (BalanceIT Canine Supplement) was required to ensure all the essential vitamins and minerals for canine adult maintenance were met. Although the exact amounts of these three ingredients varied for each dog, at least 75% of the calories were provided by the Modulen IBD liquid diet, and overall, the caloric distribution was in the region of 30.7–33.2% for protein, 32.7–33.9% for fat, and 34.0–35.3% for carbohydrate on a metabolizable energy basis for all five dog formulations. 

### 2.3. Preparation of the Diet

The diet was prepared using cleaned equipment and bottled water according to the package instructions. All three ingredients (the Modulen IBD liquid diet, whey powder, and multivitamin and mineral blend) were weighed using a gram scale and combined in a clean bowl. Bottled water was added in 25 to 50 mL increments until the slurry reached a consistency that was considered to easily pass via the esophagostomy feeding tube. The final volume of the slurry was measured, and the kcal/mL was calculated. The slurry was prepared daily and divided into four syringed feeds and refrigerated until needed. 

### 2.4. In-Hospital Feeding and Monitoring

Feeding commenced the evening of the procedure of the gastrointestinal endoscopy and esophagostomy feeding tube placement once the dog was suitably recovered from the general anesthetic. The feeding was commenced on day 1 as 25% of the resting energy requirement (RER) using the exponential equation of 70 multiplied by the dog’s bodyweight in kilograms raised to the power of 0.75, divided into 4 feeds over 24 h. This was increased by 25% of the RER increments every 24 h if the dog tolerated the diet (showed no signs of regurgitation, vomiting, or lip smacking) or exacerbation of gastrointestinal signs, such as diarrhea. If the dog showed any signs of intolerance or worsening of signs, then the amount of slurry offered was maintained for another 24 h and the signs were reassessed or decreased by 25% of the RER depending on the severity of the intolerance or worsening of the signs. If the dog was not tolerating 25% of the RER, then this was decreased to 10% of the RER and reassessed 24 h later. All feeds were exclusively completed with the Modulen IBD liquid diet. No other treats or foods were administered and multiple signs were placed on the dog’s kennel and treatment sheet to prevent inadvertent feeding. Once the feeds reached 75% to 100% of the RER, Purina Pro Plan Veterinary Diets HA Canine dry food was offered to gauge appetite, and voluntary oral intake was recorded. 

The slurry was administered via esophagostomy tube manually at a rate of approximately 1 mL per minute. The slurry was warmed for 10–15 min at room temperature, followed by gently warming in a water bath of warm water prior to administration, if needed. The esophagostomy feeding tube was flushed with 5 mL of tap water before and after each feed. 

### 2.5. Assessment of Tolerance to Diet, Effect on Gastrointestinal Signs, and Biochemical Parameters

Tolerance of the diet was closely assessed by monitoring the dog for any signs of nausea (hypersalivating or lip smacking), regurgitation, vomiting, or restlessness. This was assessed throughout hospitalization when the dog was receiving the Modulen IBD liquid diet. Written notes following every feed were made and recorded, even if the dog showed no signs. 

Throughout the hospitalization period, stool quality and the presence of any gastrointestinal signs were recorded. Appetite was assessed only after the dog was receiving at least 75–100% of the RER of the Modulen IBD liquid diet by offering Purina Pro Plan Veterinary Diets HA Canine dry food at this stage and recording voluntary intake. 

Blood was collected by venipuncture 2–3 days following the initiation of the Modulen IBD liquid diet for a standard biochemistry panel to allow for comparisons of serum albumin, globulin, cholesterol, and C-reactive protein (CRP) concentrations to admission (pre-Modulen IBD liquid diet) values. 

The Modulen IBD liquid diet was discontinued once the dog was voluntarily consuming Purina Pro Plan Veterinary Diets HA Canine dry food reliably. 

### 2.6. Statistical Analysis

The related-samples Wilcoxon signed rank test was used to determine if there were any significant differences in serum albumin, globulin, cholesterol, and CRP concentrations 2–3 days following initiation of the Modulen IBD liquid diet compared to admission (pre-Modulen IBD liquid diet) concentrations for the five dogs. This was performed using the IBM SPSS (Statistical Product and Service Solutions) version 26 statistical software program. Statistical significance was placed at *p* < 0.05. The median, range, and percentage change were calculated using Microsoft Excel for Mac Version 16.57. 

### 2.7. Ethical Approval and Owner Consent

The Royal Veterinary College granted ethical approval for the study (URN 2019 1912–3). Written informed consent for participation in the study was obtained from all owners of the dogs.

## 3. Results

### 3.1. Study Dogs

Fourteen dogs were eligible for inclusion; however, seven were excluded: two due to concurrent liver disease, two due to the primary investigator not being informed of the case for enrollment, one due to concurrent pancreatitis and liver disease, one due to duodenal small cell lymphoma, and one due to acute blood loss from a gastrointestinal ulcer as the cause for the panhypoproteinemia. Two dogs were also excluded as they were started on the Modulen IBD diet with concurrent glucocorticoids, as their serum albumin concentration was considered severe (<12 g/L). Five dogs met the inclusion criteria. 

The five dogs had a median (range) age of 5.8 years (3–10.9) and consisted of two intact males, two neutered males, and one female neutered dog. The breeds consisted of an English bulldog, a cocker spaniel, a crossbreed, a beagle, and a Yorkshire terrier. The presenting clinical signs included vomiting and diarrhea in four dogs and diarrhea in one. The median duration of gastrointestinal signs was 2 months with a range of 1 week to 3 years. The median (range) body weight was 9.05 kg (7.5–26.1) and the median (range) body condition score was 2/9 (range 2–5). Prior to referral, two dogs received one injection of dexamethasone one day prior to referral and four dogs received antimicrobials (metronidazole (*n* = 4) and oxytetracycline (*n* = 1)), though none of these medications were ongoing or continued at the time of referral. Two dogs previously received diet trials with a commercial hydrolyzed protein diet. 

The following investigations were performed for all five dogs: complete blood count, serum biochemistry panel, serum vitamin B12, transabdominal ultrasound, and upper and lower gastrointestinal endoscopy. Four of the five dogs also had urinalysis, basal cortisol, and fecal parasitology performed. Four dogs were empirically dewormed prior to referral [milbemycin oxime and praziquantel (*n* = 1), milbemycin oxime (*n* = 1), praziquantel (*n* = 1), and fenbendazole (*n* = 1)], two had fecal cultures performed, one had serum pancreatic lipase immunoreactivity, and one had a bile acid stimulation test. 

The median (range) serum albumin and vitamin B12 concentrations were 19.4 g/dL (14.8–22.1) and 361 ng/L (190–459), respectively. All 5 dogs were diagnosed with chronic inflammatory enteropathy; 3 had lymphoplasmacytic and neutrophilic duodenitis and 2 lymphoplasmacytic duodenitis, 3 had evidence of lymphangiectasia on duodenal histopathology. Four dogs had ileal histopathology performed; 2 had lymphoplasmacytic and neutrophilic ileitis and 2 lymphoplasmacytic ileitis, 2 had evidence of lymphangiectasia or lacteal dilation on ileal histopathology. All dogs had colonic histopathology performed: four had lymphoplasmacytic and neutrophilic colitis and one had plasmacytic colitis. With regards to the neutrophilic infiltration, for the duodenum, this was moderate in three; for the ileum, this was moderate in two; and for the colon, this was mild in one, mild to moderate in one, moderate in one, and moderate to marked in one. One dog was subsequently diagnosed with primary hyperparathyroidism at the same visit based on ionized hypercalcemia and high-normal parathyroid hormone (PTH) concentration (ionized calcium 1.66 mmol/L, reference range 1.25–1.45; PTH 4.0 pmol/L, reference range 0.5–5.8; and PTH-related protein 0.0 pmol/L, reference range 0.0–1.0). The median (range) canine chronic enteropathy clinical activity index [17] (CCECAI) for all dogs was 14 (9–18). 

Treatment at discharge included Purina Pro Plan Veterinary Diets HA Canine dry food and prednisolone for four out of the five dogs, and for the one remaining dog, it included a combination of the Purina HA diet with Hill’s Prescription Diet z/d Canine dry food as the dog eventually preferred this combination during hospitalization. For one out of the four dogs treated with diet and prednisolone, treatment also included metronidazole. 

### 3.2. Diet Formulation

The diet for all five dogs was formulated to ensure the minimum requirements and/or recommended allowances of all essential nutrients met those established by the National Research Council 2006 profiles for canine adult maintenance on an energy basis. The diet formulation for all five dogs was comprised of the same three ingredients: Modulen IBD liquid diet, commercial whey powder isolate, and BalanceIT Canine Supplement. The Modulen IBD liquid diet provided between 77–80% of the total calories for all five formulations. The median (range) percentage of protein, fat, and carbohydrate on a metabolizable energy basis for all five formulations was 32% (30.7–33.2), 33.3% (32.7–33.9), and 34.7% (34.0–35.3), respectively. 

### 3.3. Tolerance to Diet

All Modulen IBD liquid diet feeds at 25% of the RER were well-tolerated in three of the five dogs. For one of the dogs, the first 25% RER feed was associated with lip smacking, while the rest of the feeds were well-tolerated. For the one remaining dog, regurgitation into the mouth followed by swallowing was seen for one of the feeds; however, this resolved after the dog was started on omeprazole and ondansetron. 

Two out of the five dogs tolerated all Modulen IBD liquid feeds at 50% of the RER. For one of the dogs, two feeds were associated with lip smacking, which resolved when administration was slowed. A second dog had a small amount of regurgitation following the first feed at 50% RER and none after this. This same dog also showed some lip smacking with another 50% RER feed, which settled when administration was slowed. The one remaining dog belched once during one 50% RER feed and tolerated the rest of the feedings well. 

Four out of five dogs tolerated all Modulen IBD liquid feeds at 75% of the RER. The one remaining dog showed lip smacking with two feeds at 75% RER; however, on both occasions, this resolved once administration was slowed. 

Only three out of the five dogs received the Modulen IBD liquid diet at 100% of the RER, as the remainder of the dogs had very good to ravenous appetites and ate Purina Pro Plan Veterinary Diets HA Canine dry food well when offered at the 75% RER Modulen feeds. Of these three dogs, two tolerated all feeds at 100% of the RER. The one remaining dog demonstrated intermittent lip smacking with one feed, which was reduced when administration was slowed. 

### 3.4. Effect of Diet on Appetite and Gastrointestinal Signs during Hospitalization

All five dogs presented with anorexia, necessitating the placement of an esophagostomy feeding tube at the time of the gastrointestinal endoscopy. All five dogs voluntarily ate Purina Pro Plan Veterinary Diets HA Canine dry food prior to the commencement of glucocorticoid and after receiving the Modulen IBD liquid diet at 75% of the RER (*n* = 3) or 100% of the RER (*n* = 2). For all five dogs, appetite was reported to be very good to ravenous. Once all five dogs voluntarily consumed the Purina Pro Plan Veterinary Diets HA Canine dry food diet reliably, they received this diet exclusively, except for one dog, which received this diet with Hill’s Prescription Diet z/d Canine dry food, as the dog eventually preferred this combination during hospitalization.

The dogs were hospitalized for a median (range) of 5 days (4–8) following initiation of the Modulen IBD liquid diet. None of the five dogs had any episodes of vomiting during hospitalization after initiating the Modulen IBD liquid diet. Only two of the five dogs had episodes of regurgitation that were independent of feeding during hospitalization after initiating the Modulen IBD liquid diet. One dog had three episodes of regurgitation: once when feedings were at 25% of the RER and twice when at 50% of the RER, and the second dog had a small amount of regurgitation once when feeding was at 50% of the RER. 

For the five dogs, the effect of feeding the Modulen IBD liquid diet on stool frequency and consistency during hospitalization was as follows: for one dog, only one stool was passed when the dog was receiving 75% of the RER, and this was reported to be soft formed and light brown in color with no blood, mucus, straining, or excess volume. A second dog passed no stools during hospitalization after the diet was initiated. For a third dog, when receiving 25–50% of the RER feeds, watery stool with fresh blood and mucus was being passed up to eight times per day. This dog was then reported to pass no stools once feeds reached 75 to 100% of the RER. A fourth dog passed liquid bloody stool three times when receiving 25% of the RER feeds, which increased to six times per day when receiving 50% of the RER feeds, but then improved from projectile to smaller amounts and returned to a normal color with mucus, but no blood, at 75% of the RER feeds. The frequency at this time also improved to three times per day and the consistency further improved to soft and semi-formed. For the remaining dog, the diet did not help to improve frequency or consistency of the stool, and the dog continued to pass stool between seven to nine times per day when receiving 25% to 100% of the RER feeds. The consistency varied from watery to jelly-like and back to watery during this time. The stool was initially green, then bloody with mucus. This one dog received metronidazole in-hospital, as fecal consistency and frequency were not improving, and this medication was continued after discharge from the hospital. 

### 3.5. Effect of Diet on Selected Biochemical Parameters

Serum biochemistry was performed on all dogs 2–3 days after initiating the Modulen IBD liquid feeds. For the five dogs, three (60%) had stable or improved serum albumin concentrations (median % increase: 10.3, range: 0–31.1), four (80%) had improved or normalized serum globulin concentrations (median % increase: 12.9, range: 5.1–66.2), four (80%) dogs had improved or normalized serum cholesterol concentrations (median % increase: 31.5, range: 4.8–63), and three had improved serum CRP concentrations (median % decrease: 7.4, range: 7.4–80.9). 

The related-samples Wilcoxon signed rank test showed no significant differences in the selected biochemical parameters, both pre- and post-Modulen IBD liquid diet, for all dogs (*p* > 0.080). The results are presented in Table 1.

## 4. Discussion

The Modulen IBD liquid diet is a prescription diet intended for humans with inflammatory bowel disease (IBD), with several studies proving its therapeutic efficacy [13,14,15,16]. However, to our knowledge, this diet has not been previously investigated clinically in dogs. Therefore, our study sought to assess the tolerance to this diet and the short-term effect on gastrointestinal signs and selected biochemical parameters during hospitalization in dogs with inflammatory PLE. 

Inflammatory PLE in dogs has a guarded prognosis, with disease-associated death reported in approximately half of the cases [1,2,18]. Therefore, a more efficacious treatment is needed to help improve the prognosis in these dogs. Diet is integral to the treatment of dogs with inflammatory PLE, with multiple studies showing its efficacy [2,19,20,21,22]. However, it is unknown what dietary composition and macronutrients are ideal for treatment. So far, most of the studies have centered on the effect of low-fat diets in this disease [23,24]. Therefore, further studies investigating the effects of specific diets to determine the most efficacious are needed. As in canine inflammatory PLE, diet forms an integral part of treatment in humans with IBD [25]. The Modulen IBD liquid diet is designed to be fed as the sole source of nutrition to humans with IBD, and several studies have shown that this diet, when fed exclusively, is able to resolve intestinal inflammation and induce remission in children and adults with IBD [13,14,15,16,26]. The possible causes for the reduction in intestinal inflammation include its effects on the microbiota [15,16], mucosal healing [13,15], reduction of proinflammatory cytokines [27], and improving growth and body composition [13,14,28,29]. Therefore, this specific diet was deemed as an ideal candidate to investigate further in dogs with inflammatory PLE. 

As formulated, the Modulen IBD liquid diet is relatively high in fat at 42% on a metabolizable energy basis. However, of the 23 g of fat in this diet, 6 g come from medium chain triglycerides. Medium chain triglycerides have been shown to modulate intestinal inflammation and cause less damage than long chain triglycerides in an animal model of ileitis [30], and therefore might help to reduce intestinal inflammation in canine inflammatory PLE. The Modulen IBD liquid diet also contains transforming growth factor beta (TGF-beta). This cytokine has anti-inflammatory properties in the intestinal tract [31] and plays a crucial role in maintaining tolerance against self-antigens and those derived from food and commensal bacteria [32]. Interestingly, duodenal TGF-beta mRNA expression has been shown to be significantly lower in dogs with IBD than in healthy dogs [33]. As the direct therapeutic effect of TGF beta within the Modulen IBD diet remains to be proven [34], studies are needed to specifically ascertain if this cytokine has any beneficial effects on the gastrointestinal tract in dogs with PLE. 

As being fed only the Modulen IBD liquid diet does not meet the daily requirements of all essential nutrients for canine maintenance, whey powder and a multivitamin and mineral blend were added to this diet to ensure it was complete and balanced for adult dogs. All dogs were closely monitored for worsening gastrointestinal signs when the diet was being introduced, as this may have indicated an intolerance to the whey powder or multivitamin and mineral blend. For the five dogs in our study, as the gastrointestinal signs generally improved, it was assumed that they tolerated the ingredients present in the diet. However, future studies using this diet and additional ingredients should take into account that dogs may be intolerant to some ingredients, particularly the whey powder, and therefore these dogs should be closely monitored for any indications of this so that the diet can be discontinued.

Our study showed that the Modulen IBD liquid diet was well-tolerated in all dogs, with only a few episodes of lip smacking or regurgitation in a few of the dogs; however, the former resolved or improved when the speed of administration was slowed. One study in dogs showed vomiting as a common complication following initiation of enteral feeding in dogs with hemorrhagic diarrhea [35]. As our study showed that this potential common complication was absent with the Modulen IBD liquid diet, this diet may be more advantageous compared to other enteral liquid diets fed to dogs. The largest effect of the Modulen IBD liquid diet was on the dogs’ appetites whilst hospitalized. All dogs were anorexic prior to hospitalization, necessitating the placement of an esophagostomy feeding tube. However, for all five dogs, their appetites increased, allowing the significant voluntary intake of Purina Pro Plan Veterinary Diets HA Canine dry food after receiving the Modulen IBD liquid diet at 75 or 100% of their RER (on day 3 to 4 of initiating the diet). The possible reason for this increase in hunger could be the positive effects of the diet in reducing intestinal inflammation and promoting mucosal healing. The ability of the Modulen IBD liquid diet to improve voluntary oral intake in dogs with inflammatory PLE has many advantages. Firstly, this allows the enteral feeding tube to be removed earlier, and therefore the effort of feeding via the tube and any complications associated with the tube being in place can be avoided. Secondly, more therapeutic options can be provided via the mouth, such as hydrolyzed protein diets, which may be difficult to blendarize and pass through a feeding tube due to their consistency. In addition, the potential lower palatability of hydrolyzed protein diets may make oral feeding difficult when appetite is reduced, hence potentially decreasing compliance with these diets, and therefore their effect, which has been proven in multiple studies [36,37,38,39]. Therefore, ensuring voluntary oral intake returns as soon as possible in these dogs is advantageous, and thus may make the Modulen IBD liquid diet more favorable. 

For some dogs in our study, the Modulen IBD liquid diet resulted in an improvement in stool consistency and frequency during hospitalization. Interestingly, human formulations of enteral liquid diets have anecdotally been avoided in dogs due to the concern that the increased osmolality may worsen stool consistency. However, this may be less of a concern with the Modulen IBD liquid diet, as the osmolality is lower due to the higher fat content. The reasons for the improvement in stool consistency in our study dogs are unknown but can likely be attributed to the direct effect of the diet on the intestinal mucosa. Further, the TGF-beta in the diet’s formulation might have helped to improve intestinal inflammation and mucosal healing, resulting in less diarrhea. Similarly, the higher medium chain triglyceride content might have also helped to improve intestinal inflammation. In one dog, the frequency and consistency of the diarrhea did not improve following the initiation of the diet. As inflammatory PLE in dogs is heterogenous with regards to its pathogenesis, this may explain the varied effect of this diet on the gastrointestinal signs in this dog. Further studies will help to determine which subset of dogs with inflammatory PLE is likely to benefit most from this diet, as well as the mechanisms for any beneficial effects on stool consistency and frequency. 

The presence of initial transient green stool has been noted as an effect of the Modulen IBD liquid diet in humans [40] and is due to a buildup of biliverdin, which is a likely consequence of the cessation of the normal microbial breakdown of biliverdin to stercobilin. Interestingly, in our study, this was seen convincingly in one dog. In this dog the occurrence of green stool was also present transiently at the initiation of the diet. This suggests similar effects of this diet in both species, and therefore any beneficial effects of this diet in humans with IBD, such as reduction in intestinal inflammation and increase in remission rates, may also cross over to dogs with inflammatory PLE.

The Modulen IBD liquid diet has been shown to increase serum albumin concentrations faster than corticosteroids in children with IBD [12]. A faster increase in serum albumin is desirable in dogs with inflammatory PLE so that complications such as ascites and thromboembolism can be avoided. Additionally, serum albumin concentration has been shown to be a prognostic indicator in dogs with chronic enteropathy [17], and one study showed that the normalization of plasma albumin concentrations within 50 days of initial treatment of PLE was associated with a longer survival time [41]. Therefore, treatment aimed at increasing serum albumin concentrations faster is advantageous and desirable in dogs with inflammatory PLE. In our study, we performed serum biochemistry 2–3 days after initiating the diet. In the five dogs in our study, the diet resulted in stable or improved serum albumin concentrations in three dogs 2–3 days after starting the diet. A more marked improvement may have been seen had the dogs been receiving 100% of the RER of the diet at the time of blood collection. In addition, the relatively longer half-life of serum albumin of approximately 7 days in dogs [42] and the relatively little acceleration of albumin synthesis in humans with gastrointestinal protein loss [43] may mean follow-up biochemistry following longer feeding times would potentially result in the effects of the diet being more apparent. In one dog, the serum albumin concentration decreased by at least 10%; however, this dog also had concurrent primary hyperparathyroidism that was subsequently diagnosed at the same visit, and therefore the impact of changing dietary calcium levels on subsequent serum albumin concentrations is unknown. This further highlights the need to take into account comorbidities when considering feeding with the Modulen IBD liquid diet. One of the dogs in our study had at least a 30% improvement in serum albumin following the diet. This dog eventually went into biochemical and clinical remission with dietary therapy alone, and therefore, in this dog, the Modulen IBD liquid diet may have had more of an impact. Multiple studies have shown that there is a subset of dogs with inflammatory PLE that may enter remission with dietary therapy alone [2,19,20]. Thus, further studies should aim to investigate the effects of the Modulen IBD liquid diet in this subgroup. 

The Modulen IBD liquid diet appeared to have the greatest effect on serum globulin concentrations 2–3 days after feeding this diet. Serum globulins are made up of three components: alpha and beta globulins, which are synthesized by the liver, and gamma globulins, which are synthesized by plasma cells [44]. An increase in serum globulins in our study may reflect decreased loss via the gastrointestinal tract, although serum globulin may also increase with inflammation. However, in our study, as the serum CRP followed similar trends of reduction with the increase in serum globulin, this suggests that the inflammation is an unlikely cause for the increase seen in our study. The measurement of fecal alpha-1 proteinase inhibitor before and after receiving the diet in these dogs may have helped to definitively determine the mechanism for the serum albumin and globulin changes seen [45]. In our study, the serum globulin concentration 2–3 days after receiving the Modulen diet did not reach statistical significance; however, this might have been due to our study being underpowered, as only five dogs were included in the statistical analysis. Therefore, our study justifies the need for studies utilizing a larger number of dogs with inflammatory PLE. A small number of dogs were enrolled in our pilot study in an effort to reduce the number of animals used in a research study with an unknown outcome.

The Modulen IBD liquid diet also had a positive effect of increasing serum cholesterol concentrations 2–3 days after initiating feeding. Although this was apparent for four out of the five dogs, one dog had a decrease despite improvements in serum albumin and globulin and normalization of serum CRP. This dog had lymphangiectasia on intestinal histopathology, and therefore one possibility for the decreased serum cholesterol following feeding of this diet might have been due to the higher fat content resulting in increased loss of lymph and, therefore, cholesterol via the gastrointestinal tract. However, given that the CRP had normalized in this dog and that the other dogs with lymphangiectasia or lacteal dilation on intestinal histopathology still had increased concentrations of cholesterol following the diet suggests that there might be another reason for this dog’s decrease. As stated above, the heterogenous pathogenesis of inflammatory PLE may mean that not all dogs with this condition will respond equally to dietary management, and therefore further studies are needed to help identify which subset is most likely to respond to this strategy.

Although the Modulen IBD liquid diet did not significantly reduce inflammation across the five dogs, as measured by CRP, this is likely due to dog four showing a major increase. As mentioned above, dog four was subsequently diagnosed with primary hyperparathyroidism at the same visit, which may have influenced subsequent CRP concentrations [46]. However, another possibility could be that this dog is a dietary non-responder. There are children with Crohn’s disease who are non-responders to this diet, though they are relatively small in proportion at 10–23% of those treated [47]. Therefore, it is possible that with a larger study, the percentage change in CRP will become significant, as the presence of a non-responder is likely to make a bigger difference in a small study. 

The results of our study necessitate further studies assessing the longer-term effects of the Modulen IBD liquid diet in a larger number of dogs with inflammatory PLE. Studies assessing whether this diet helps to increase biochemical and clinical remission are needed. In addition, the mechanism of action of this diet and the reason for its efficacy, as well as identifying which subset of dogs within this condition are most likely to respond, are needed. 

In addition to the limitations mentioned above, additional limitations of our study include: the Modulen IBD liquid diet was not fed as a complete diet, as this diet as fed is not complete and balanced for dogs. However, at least 75% of the total calories were provided from this diet, and therefore it is suspected that the efficacy would have been maintained. As our study was assessing short-term feeding, data on the effect of the diet on body weight was not taken into account. Therefore, future studies assessing the longer-term outcome should take this into consideration, especially as malnutrition is prevalent in this disease and the Modulen IBD liquid diet has been shown to improve body composition in children with IBD receiving this diet [13,14,28,29]. Unfortunately, no standardized scoring system for the feces was used to allow more objective data on any changes after feeding the diet. Similarly, repeat CCECAI scores were not assessed during hospitalization to allow for a measure of objective change. Hence, future studies should also account for this. Finally, our study did not include a control diet to help compare the specific effects of the Modulen IBD liquid diet against, and, as such, future studies should also focus on a group of dogs with inflammatory PLE receiving a control diet, such as a therapeutic gastrointestinal highly digestible diet.

## 5. Conclusions

In conclusion, the Modulen IBD liquid diet fed via an esophagostomy feeding tube was well-tolerated in-hospital, and resolved anorexia in all dogs with inflammatory PLE and helped to improve selected biochemical parameters in some dogs. Further studies are needed to assess the long-term effects of feeding this diet on the rate of serum albumin increase and remission in dogs with inflammatory PLE. 

## Figures and Tables

**Table 1:** Selected serum biochemical parameters for the five dogs diagnosed with inflammatory protein-losing enteropathy before (pre) and 2–3 days after (post) receiving the Modulen IBD liquid diet exclusively in-hospital without concurrent glucocorticoids. The percentage change (%) that were deemed to be beneficial are presented in bold. The *p*-value represents the results from the related-samples Wilcoxon signed rank test. CRP = C-reactive protein. RR = laboratory reference range. The * dog was subsequently diagnosed with primary hyperparathyroidism based on ionized calcium and serum parathyroid hormone (PTH) and PTH-related protein concentrations at the same visit.

Dog	Albumin (g/L) RR 26.3–38.2			Globulin (g/L) RR 23.4–42.2			Cholesterol (mmol/L) RR 3.2–6.2			CRP (mg/L)		
	Pre	Post	%	Pre	Post	%	Pre	Post	%	Pre	Post	%
1	19.8	19.4	−2.0	22.2	25.9	**+16.7**	3.00	3.80	**+26.7**	74.0	68.5	**−7.4**
2	15.5	17.1	**+10.3**	17.8	18.7	**+5.1**	2.35	1.55	−34.0	13.1	2.5	**−80.9**
3	21.7	21.7	**0.0**	23.0	25.1	**+9.1**	2.56	3.49	**+36.3**	18.6	18.8	+1.1
4 *	16.0	14.4	−10.0	22.8	22.0	−3.5	2.70	2.83	**+4.8**	4.30	71.8	+1569.8
5	14.8	19.4	**+31.1**	14.5	24.1	**+66.2**	2.00	3.26	**+63.0**	42.0	38.9	**−7.4**
*p*-value	0.581			0.080			0.176			0.686		

## Data Availability

All data is provided in the manuscript.
